# Cone-Beam Computed Tomography Laser-Guided Transthoracic Needle Biopsy for Pulmonary Lesions in a Hybrid Operating Room: Feasibility Study by an Interventional Pulmonologist

**DOI:** 10.3390/diagnostics16020226

**Published:** 2026-01-10

**Authors:** Lun-Che Chen, Po-Keng Su, Geng-Ning Hu, Shwetambara Malwade, Wen-Yuan Chung, Ling-Kai Chang, Shun-Mao Yang

**Affiliations:** 1 Interventional Pulmonology Centre, National Taiwan University Hospital, Hsin-Chu Branch, Hsinchu 300195, Taiwan; b94401048@gmail.com (L.-C.C.); andy9901105@gmail.com (G.-N.H.);; 2 Department of Internal Medicine, National Taiwan University Hospital, Hsin-Chu Branch, Hsinchu 300195, Taiwan; 3 Department of Surgery, National Taiwan University Hospital, Hsin-Chu Branch, Hsinchu 300195, Taiwan; 4 Department of Advanced Therapies, Siemens Healthcare Limited, Taipei City 11503, Taiwan

**Keywords:** percutaneous transthoracic needle biopsy, cone-beam computed tomography, pulmonary lesions

## Abstract

**Background/Objectives:** Percutaneous transthoracic needle biopsy (PTNB) using advanced navigation techniques is increasingly performed; however, pulmonologists’ experience remains limited. This study reports an interventional pulmonologist’s initial experience with cone-beam computed tomography (CBCT) laser-guided PTNB and the diagnostic performance for lesions with diameters greater than or less than 20 mm. **Methods:** We retrospectively analysed the data of patients who underwent PTNB in a C-arm CBCT-equipped hybrid operating room between July 2020 and March 2024. All patients underwent the biopsy procedure under local anaesthesia. This was preceded by an initial 3D scan for planning of the needle route, followed by coaxial needle insertion. A post-procedural scan was also performed to identify complications. **Results:** Seventy-seven patients were enrolled in the study. The median distances of the needle path from the skin to the pleura and from the pleura to the lesion were 33.4 mm and 31.7 mm, respectively. The median number of tissue samplings was 4.9 ± 1.8. The median operating room duration was 51.5 ± 25.7 min, respectively. The median total dose area product was 8485.4 ± 5819.9 µGym^2^. The sensitivity and specificity of our study findings were 93.3% (56/60) and 100%, while the accuracy was 94.8% (73/77). The overall complication rate was 13%. **Conclusions:** PTNB procedure by pulmonologists is a feasible and safe, single-operator workflow in a hybrid operating room. It can be performed under CBCT laser guidance with a similar diagnostic yield, acceptable radiation exposure and procedure duration, and minimal or manageable complications.

## 1. Introduction

Percutaneous transthoracic needle biopsy (PTNB) of lung lesions is a common procedure with a high diagnostic yield and an acceptable complication rate [1,2,3]. It can aid in the diagnosis of peripheral lung lesions (PPLs) and is a minimally invasive procedure for collecting adequate tissue for diagnosis and related biomolecular tests in cases of suspected malignant lesions [4,5]. Despite the increasing popularity and widespread adoption of electromagnetic navigation, virtual bronchoscopy, and endobronchial ultrasound by pulmonologists and the higher diagnostic yield of >90% for these techniques, experience in performing PTNB is limited [6,7]. Performing PTNB with computed tomography (CT) and CT fluoroscopy guidance has been increasingly utilised and continues to evolve for the diagnosis of small lung lesions [1,8].

PTNB using conventional CT is a well-established and widely used technique. It has minor complications and a low major complication rate [9,10]. Its feasibility and safety have also been established through extensive research [11,12,13,14]. However, it is usually performed by interventional radiologists, and the involvement of pulmonologists has not been extensively documented. Conventional fan-beam CT-guided PTNB has several limitations. First, real-time monitoring is not performed during the puncture. The needle can be advanced only based on the predefined trajectory determined from the initial CT scan [15,16,17]. In addition, the needle may miss the target if the patient is not breathing appropriately. This can result in multiple puncture attempts and increase the risk of lung parenchymal injury. It can also prolong the procedure and increase radiation exposure. Second, the needle placement is restricted by the axial imaging plane of the CT scanner and the small gantry bore [18]. Therefore, achieving an ideal puncture path under conventional CT guidance may be challenging in some cases.

A novel technique for PTNB guidance has been under development with the emergence of cone-beam computed tomography (CBCT). It combines needle path planning with three-dimensional (3D) CBCT imaging. CBCT systems also offer real-time monitoring of PTNB procedures and greater flexibility for the orientation of the detector system around the patient than conventional CT systems [2,19,20,21]. PTNB procedures supported by advanced 3D needle guidance systems can be performed in a sterile workspace with flexible system angulation and immediate fluoroscopic feedback [22].

Our centre initiated these procedures in the hybrid operating room (HOR) in 2020. The HOR was established to facilitate single-stage interventions and allow for a schedule of cost sharing. The system facilitates efficient planning and guidance for successful lung marking, even for those with limited procedural experience, because CBCT provides image guidance for both bronchoscopy localisation and percutaneous biopsy [23]. Intraoperative confirmation scans and image reconstruction can also help thoracoscopic surgeons perform satisfactory percutaneous localisation and resection. The involvement of interventional pulmonologists, thoracic surgeons, and radiologists can facilitate individualised planning, intervention, and intraoperative complication management during biopsy [23]. The pulmonologist should consider factors such as lung ventilation, airway evaluation, and procedure-related risk factors, including needle-related adverse events, especially in cases of concomitant biopsy during ablation procedures in the HOR [24]. We adopted the same approach and settings for the biopsy procedures in the HOR to facilitate navigation to difficult-to-access or small lesions in complicated cases.

We report our initial experience with PTNB performed by interventional pulmonologists with CBCT laser guidance. We also report the details of the biopsy procedure and compare the diagnostic performance for PPLs with maximal diameters greater than or less than 2 cm.

## 2. Materials and Methods

### 2.1. Patients

Seventy-seven consecutive patients who underwent iGuide percutaneous core needle biopsy for lung lesions at the National Taiwan University Hospital, Hsinchu Branch, from July 2020 to March 2024 were recruited for this study. The procedures were performed in the HOR equipped with a robotic C-arm CBCT system (ARTIS pheno; Siemens Healthineers AG, Erlangen, Germany). The data were acquired from the institutional clinical database and the medical records of the teaching hospital. The Institutional Review Board of the National Taiwan University Hospital (202310003RINC) approved this study.

### 2.2. Workflow

**Figure 1:**
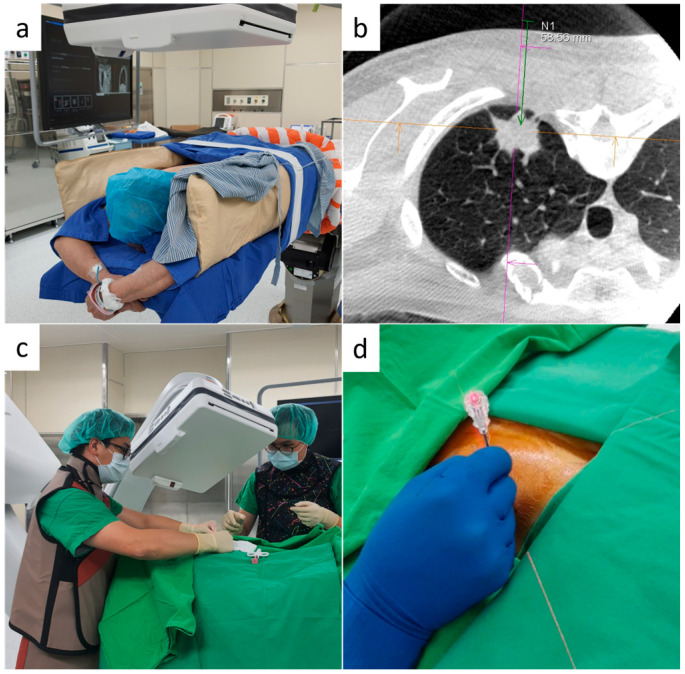
Patient preparation and settings for the iGuide biopsy procedure. (**a**) The patient was fixed in the prone position using a vacuum bean bag. (**b**) The lesions in the posterior region are visible in the CBCT images acquired in the same position (iGuide toolbox for lesion segmentation). (**c**) Interventional pulmonologists use the flat-panel detector of the C-arm to ensure the correct positioning of the laser beam. (**d**) The laser beam was projected onto the patient’s skin to guide the needle entry point. The needle was inserted into the chest wall after confirming that the centre of the laser cross was aligned with the outer tip of the needle.

All the enrolled patients underwent the biopsy procedure under local analgesia. Based on the location of the lung lesion. They were positioned in the supine, prone, or lateral decubitus position for the optimal access route for needle insertion and subsequently placed on the table with a vacuum positioner (Figure 1a).

### 2.3. iGuide Planning

An initial scan with a 4 s acquisition protocol (4s DynaCT Body) was performed under end-inspiratory breath-hold, which was set up by clamping the endotracheal tube using a haemostat once the ventilator bellows provided the targeted tidal volume. The scanned images were transferred to a nearby workstation, Syngo X-Workplace (Siemens Healthineers AG, Erlangen, Germany), and the needle access path was laid out in the isotropic data set using the Syngo Needle Guidance (Siemens Healthineers AG, Erlangen, Germany). The needle path was defined by marking the entry and target points of the needle (Figure 1b). These points were subsequently projected onto the patient’s skin using a laser cross beam integrated with the C-arm CBCT system.

### 2.4. Needle Advancement and Biopsy

The pulmonologist performed all procedures, guided by CBCT-fluoroscopy with the cross-laser beam projected by the C-arm flat-panel detector (Figure 1c). The 17G coaxial needle was inserted through the laser-marked point on the skin. The needle orientation was adjusted until the needle tip and hub were aligned with the laser crossbeam (Figure 1d).

**Figure 2:**
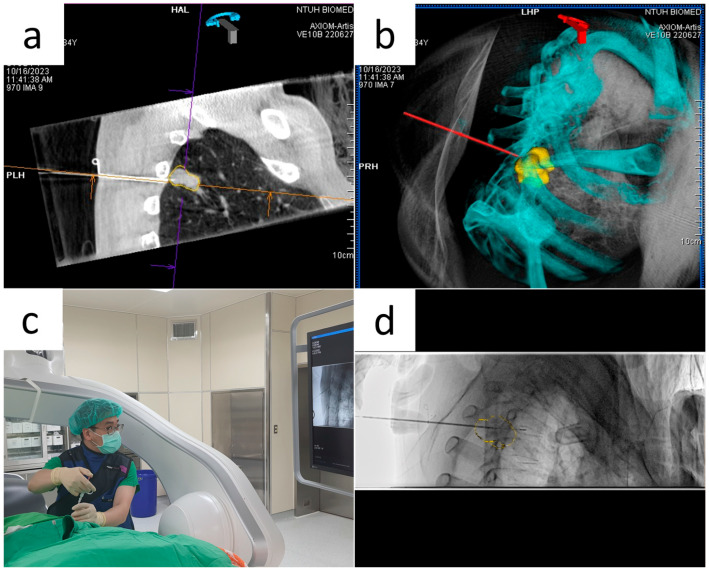
Needle insertion and confirmation during the biopsy procedure. (**a**) CBCT scan after initial needle insertion into the chest wall to ensure that the needle is on the planned route (iGuide toolbox used for lesion segmentation). (**b**) 3D reconstruction of the whole thorax to visualise the spatial relationships between the needle and the surrounding bony structures and enable rotation and rectification of deviations. The yellow mass shown is a 3-D segmentation of the target lesion. (**c**) The C-arm was turned to the progression view for further needle insertion under fluoroscopic guidance. (**d**) The needle is on target, as observed under the progression view on the monitor. The yellow contour indicates the lesion.

CBCT was repeated to determine the need for needle repositioning after the needle was inserted into the chest wall along the laser crossbeam. Its position was stabilised (Figure 2a). The spatial relationship between the needle and the bony parts of the chest wall was visualised using 3D image reconstruction (Figure 2b). This facilitated the repositioning of the needle. The needle was advanced under fluoroscopy after the skin entry point, and orientation was determined. CBCT was subsequently performed to confirm its final position. The stylet of the 17G coaxial needle was removed after confirming that the needle tip was close to the target lesion. An 18-G core biopsy needle was inserted. The inner stylet with the specimen notch was manually advanced under fluoroscopic guidance (Figure 2c). Augmented fluoroscopy with lesion contour visualisation on real-time fluoroscopy can be used, especially for fluoroscopically invisible lesions (Figure 2d). On average, 3–6 tissue samples were taken. The coaxial introducer was removed after sufficient tissue samples had been obtained. Post-procedure CBCT images were acquired to identify procedure-related complications.

### 2.5. Data Collection and Statistics

We collected clinical data, including patient characteristics, features of the pulmonary lesions, and procedural details. The procedure duration was measured from the start of needle insertion to the last CBCT scan. The global operating room duration was determined from the time the patient entered the HOR to the time of leaving. The total accumulated radiation exposure dose, expressed as the dose-area product, was retrospectively calculated from data stored in the ARTIS workstation (Syngo Workplace). We also assessed the complication rates and final pathological diagnoses. The diagnostic yield was calculated as the proportion of tests with a definitive diagnosis among all attempted tests. Descriptive statistics for the continuous data are summarised as means and standard deviations, and those for the categorical data are summarised as counts and percentages. The technical success rate, sensitivity, specificity, and accuracy were determined. The diagnostic accuracy was calculated to determine how accurately a test can distinguish diseased from non-diseased patients, as aligned with the key metrics -sensitivity and specificity. Univariate analysis was conducted using Fisher’s exact test, Welch’s *t*-test, and the chi-square test of independence. The success of the procedure was defined as the proportion of lesion samples that yielded a definitive pathological diagnosis. We used the STROBE reporting guideline to draft this manuscript and included the STROBE checklist in the Supplementary Materials.

## 3. Results

### 3.1. Patient Characteristics

iGuide percutaneous core needle biopsy was performed in the HOR for 77 lesions in 77 patients. The patient and lesion characteristics are provided in Table 1. Among the patients, 46.8% were female, and 53.2% were male. Of the 77 lesions, 69 (89.6%) were solid, and eight (10.4%) were subsolid. The median size and depth of the lesions were 32.5 ± 19.2 mm and 12.6 ± 9.8 mm, respectively.

### 3.2. Biopsy Procedure

Table 2 provides the details of the biopsy procedure. The procedure was performed in the supine, lateral, and prone positions for 29 (37.7%), 6 (7.8%), and 42 (54.5%) patients, respectively. The median distances of the needle path from the skin to the pleura and from the pleura to the lesion were 33.4 mm and 31.7 mm, respectively. The median number of tissue samplings was 4.9 ± 1.8. The median total procedure and operating room durations were 20.1 ± 11.2 and 51.5 ± 25.7 min, respectively. The median number of DynaCT scans performed was 3.6 ± 1.3, and the total dose area product was 8485.4 ± 5819.9 µGym^2^ (Effective dose 13.57 ± 9.31 mSv). The procedure-related complications included pneumothorax (10.4%), haemoptysis (1.3%), and haemothorax (1.3%). The overall complication rate was 13%.

### 3.3. Diagnostic Details

The details of the diagnosis of pulmonary lesions are described in Table 3. Of the lesions that were biopsied, 63 were diagnosed as malignant (*n* = 56) or benign (*n* = 7). Among them, 46 were larger than 20 mm in size, while 17 had sizes of ≤ 20 mm. Malignant diagnoses for lesions with sizes > 20 mm included lung adenocarcinoma (*n* = 27), squamous cell carcinoma (*n* = 2), non-small cell carcinoma (*n* = 10), small cell carcinoma (*n* = 1), and metastatic tumour (*n* = 2). For lesions with sizes ≤ 20 mm, the malignant diagnoses included lung adenocarcinoma (*n* = 6), non-small cell carcinoma (*n* = 3), and metastatic tumours (*n* = 5). Lesions with sizes > 20 mm diagnosed as benign included granulomatous inflammation (*n* = 2), neurogenic tumour (*n* = 1), and sclerosing pneumocytoma (*n* = 1); while lesions with sizes ≤ 20 mm included granulomatous inflammation (*n* = 2) and hamartoma (*n* = 1). Fourteen lesions were not diagnosed: six with sizes > 20 mm and five with sizes ≤ 20 mm were characterised by chronic inflammation, and one with a size ≤ 20 mm was characterised by atypical cells. The remaining two lesions measuring ≤ 20 mm each yielded no representative samples. Thus, the overall diagnostic yield for lesions with sizes > 20 mm was 88% (46/52), while that for lesions with sizes ≤ 20 mm was 68% (17/25).

Table 4 compares the characteristics and findings of the diagnostic and non-diagnostic lesions. The average size of the diagnostic lesions found on biopsies was 34.5 ± 19.8 mm, which was significantly larger than that of the non-diagnostic lesions (21.9 ± 12.4 mm). The average lesion depth of the diagnostic lesions from the pleura was 12.9 ± 9.7 mm, and the non-diagnostic lesions were 11.2 ± 11.2 mm deep from the pleura. Among the lesions with diagnostic findings on biopsy (*n* = 65), 93.8% (61/65) were solid lesions, while 6.2% (4/65) were sub-solid lesions. On the other hand, non-diagnostic lesions (*n* = 12) included 66.7% (8/12) solid lesions and 33.3 (4/12) sub-solid lesions. There were significant differences between the characteristics of the diagnostic and non-diagnostic lesions.

The sensitivity and specificity of our study findings were 93.3% (56/60) and 100%, respectively, while the accuracy of our study was 94.8% (73/77).

## 4. Discussion

This study demonstrates the technical feasibility of PTNB under CBCT and laser guidance in the HOR. CBCT provides a cross-laser for 3D visualisation and enables precise planning of the needle route axis and needle pathway. This contrasts with conventional CT, which uses a single-axis line laser. CBCT may require longer preparation for AP/lateral view adjustments and collision checks, as well as longer scan durations. In contrast, conventional CT lacks a cross laser. Therefore, it can be set up more quickly, perform multiple scans within a shorter duration, and eliminate the need for collision checks because the patient is already inside the arm. However, conventional CT is associated with a higher radiation dose and may increase the risk of complications. This is attributable to the reduced accuracy of the single laser axis, which necessitates multiple needle adjustments [15,16]. Performing the procedure under CBCT and cross-laser guidance and planning it using iGuide requires fewer needle adjustments and is associated with a lower or negligible risk of complications.

A key factor in the success of the biopsy procedure is obtaining high-quality, optimal reference images from start to finish. The accuracy of the images depends on the immobilisation of the patient, as well as the target organ. We prevented patient movement by using the vacuum positioner. This vacuum bag helped restrict body movement and ensured that the region of interest remained unchanged during the first CBCT scan and subsequent real-time fluoroscopy.

Another important aspect of the CBCT laser-guided biopsy technique was the use of augmented fluoroscopy, which is important for controlling the depth of the biopsy needle under progression view [25]. We used the bulls-eye view under augmented fluoroscopy to align the needle with the centre of the laser cross on the skin. AF is important for confirming that the needle tip is on the lesion surface after switching to the progression view and further penetrating the pleura. It enables the visualisation of small lesions that may not be visible on standard fluoroscopy. It also facilitates real-time fine adjustments [25] and multiple biopsy attempts for different parts of a large lesion. AF can be adjusted to different angles to facilitate the visualisation of different parts of the lesion.

The pulmonologists at our hospital performed the biopsies. They are more familiar with needle punctures, real-time imaging, and ultrasound procedures than radiologists, who have more experience with conventional CT-guided procedures. However, they are not familiar with the CT-guided procedure. iGuide can help pulmonologists overcome the learning curve when using the HOR. Most pulmonologists do not have access to HOR and rely on 2D fluoroscopy. However, bronchoscopy suites have recently begun providing CBCT for percutaneous procedures, which can be used with iGuide and cross-laser tools. For interventional pulmonologists who lack training for conventional CT-guided percutaneous procedures, CBCT with a laser-guided technique could be an applicable and accessible alternative. The diagnostic yield in our study was significantly higher for lesions of size >20 vs. ≤ 20 mm (88% vs. 68%). Lower diagnostic yield in smaller lesions (<20 mm) and chronic inflammation may contribute to non-diagnostic findings in these lesions.

Only one study (Jeon et al., 2018) reported the use of an additional laser guidance system for percutaneous biopsy (Table 5) [26]. The laser system had to be set up manually. The CBCT C-arm system with integrated laser guidance was convenient to use because it required no additional setup. It was automatically positioned and aligned to the lesion based on the software settings. The sensitivity in our study was lower than that reported by most previous studies with a larger lesion size of >33.8 mm. For instance, Cheng et al. [27], Ahn et al. [7], Jeon et al. [26], and Fior et al. [5] reported a sensitivity between 94% and 97%, while Soidan et al. [8] reported a lower sensitivity of 91.5%. However, the specificity was higher or similar (100%) to most of the reported values, while the specificity was higher or similar (100%). The accuracy of the study (94%) was within the range reported in most studies (92–98.8%) performed by interventional radiologists [5,15,19,27,28,29,30]. The diagnostic yields (88% and 68%) fell within previously reported ranges, although direct statistical comparison was not performed because the external datasets were unavailable. Our complication rate for pneumothorax (10.4%) was lower than those reported by Soidan et al. [8], Yang et al. [15], and Fior et al. [5], with high lesion sizes (>33.8 mm); these studies were mostly performed by radiologists, except for that by Jeon et al. [26], which was performed by a pulmonologist; haemothorax and haemoptysis were also lower than those reported in other studies [2,5,8,15,26,27,28,29,30]; these studies reported 2–17.3% of haemothorax and haemoptysis, while ours reported only 1.3%. Our procedure time (21.1 min) was longer than that of Jiao et al. (12.84 min) [19], Lee et al. (14.9 min) [2], Choo et al. (10.5 min) [29], Huang et al. (12 min) [28], and Yang et al. (15 min) [15], all of whom used CBCT systems. This longer duration may be attributed to several factors, including the time for arranging the C-arm during each CBCT scan and procedure being conducted by interventional pulmonologists, who were less familiar with the set-up. Furthermore, the overall time spent in the room was prolonged due to the inclusion of the comprehensive HOR set-up and planning, while the active biopsy was performed in an efficient manner. This balance is optimal in the HOR environment as it allows immediate utilisation of the same environment and imaging for subsequent therapeutic applications.

This study had some limitations. Firstly, it was retrospective and nonrandomised and had a small sample size. Secondly, it included patients with small and large tumours, but the iGuide biopsy approach is suitable for large tumours. Further studies could focus on small tumours and compare the findings with those of previous series. Thirdly, selection bias may have occurred. We prioritised the transbronchial technique for cases in which the lesion was accessible via bronchoscopy and used the percutaneous approach for those in which the lesion was not accessible via bronchoscopy. Lastly, a direct quantitative comparison between a pulmonologist-controlled procedure and a radiologist-controlled procedure was not possible because no radiologist-performed biopsies were available at our institute. Thus, the study’s purpose was limited to reporting early-phase experience and characterising training-transfer potential.

This initial experience demonstrates that interventional pulmonologists can safely and effectively perform PTNBs using CBCT with iGuide and laser guidance in HOR facilities. The set-up with a pulmonologist can help create a more streamlined, single-operator diagnostic–therapeutic pathway. This workflow can lead to rapid treatment planning for lesions that may not be amenable to bronchoscopic sampling. It can result in a high diagnostic yield, acceptable radiation exposure and procedure duration, reduced length of hospital stay, and minimal or well-managed complications. The workflow can help overcome the learning curve and enable ease of adoption and successful performance by those with minimal experience. The use of a coaxial needle and needle retrieval technique can help reduce the risk of pneumothorax. The HOR settings facilitate immediate detection and help prevent the progression of complications through gas aspiration, drainage insertion, and other interventions.

## Figures and Tables

**Table 1:** Characteristics of patients and pulmonary lesions.

Variables	Values
Total patients	77
Sex Female	36 (46.8%)
Age (y)	65.2 ± 13.8
ASA class	
I or II	68 (88.3%)
III	9 (11.7%)
Height (cm)	161.0 ± 9.5
Weight (kg)	60.4 ± 10.4
History of smoking	15 (19.5%)
Lesion appearance	
Subsolid	8 (10.4%)
Solid	69 (89.6%)
Location	
Right upper lobe	25 (32.5%)
Right middle lobe	5 (6.5%)
Right lower lobe	15 (19.5%)
Left upper lobe	16 (20.8%)
Left lower lobe	13 (16.9%)
Chest wall or pleura	2 (2.6%)
Anterior mediastinum	1 (1.3%)
Lesion size (mm)	32.5 ± 19.2
Lesion depth (mm)	12.6 ± 9.8

Continuous data are shown as mean/standard deviation (SD). ASA class: American Society of Anesthesiologists physical status classification.

**Table 2:** Details of biopsy procedure.

Variables	Values
Patient position	
Supine	29 (37.7%)
Lateral	6 (7.8%)
Prone	42 (54.5%)
Needle path distance	
From skin to pleura (mm)	33.4 ± 12.7
From pleura to lesion (cm)	31.7 ± 19.9
Number of tissue samplings	4.9 ± 1.8
Total procedure time (min)	20.1 ± 11.2
Total operating room time (min)	51.5 ± 25.7
Radiation	
Number of DynaCT scans	3.6 ± 1.3
Total dose area product (µGym^2^) *	8485.4 ± 5819.9
Complications	13% (10/77)
Pneumothorax	8 (10.4%)
Haemoptysis	1 (1.3%)
Haemothorax	1 (1.3%)

Continuous data are shown as median (interquartile range, IQR) and mean ± standard deviation, and categorical data as number (%). * (Effective dose 13.57 ± 9.31 mSv).

**Table 3:** Diagnosis of the pulmonary lesions.

Histopathologic Finding	>20 mm (*n* = 52)		≤20 mm (*n* = 25)	
	*n*	Details of Final Diagnosis	*n*	Details of Final Diagnosis
Diagnostic *	46 (88%, 95% CI: 77.0–94.6%)		17 (68%, 95% CI: 48.4–82.8%)	
Malignant				
Lung adenocarcinoma	27		6	
Squamous cell carcinoma	2		0	
Non-small cell carcinoma	10		3	
Small cell carcinoma	1		0	
Metastatic tumour	2		5	
Benign				
Granulomatous inflammation	2		2	
Neurogenic tumour	1		0	
Hamartoma	0		1	
Sclerosing pneumocytoma	1		0	
Non-diagnostic	6		8	
Chronic inflammation	6	Lung cancer (*n* = 2) Suspect TB (*n* = 1) Sclerosing pneumocytoma (*n* = 1) Improved (*n* = 1) Unchanged (*n* = 1)	5	Metastatic tumour (*n* = 1) Improved (*n* = 1) Unchanged (*n* = 1) IRAE (*n* = 1) Expired (*n* = 1)
Atypical cell	0		1	Unchanged (*n* = 1)
No representative samples	0		2	Metastatic tumour (*n* = 1) Interstitial fibrosis (*n* = 1)

* Fisher’s exact test: *p* value = 0.048.

**Table 4:** Comparison between diagnostic and non-diagnostic lesions.

	Diagnostic (*n* = 65)	Non-Diagnostic (*n* = 12)	*p* Value
Age	64.9 ± 14.6	67.3 ± 8.7	$0.446^{\;a}$
Female sex	30 (46.2%)	7 (58.3%)	$0.536^{\;b}$
BMI (kg/m^2^)	23.2 ± 4.3	23.6 ± 2.9	$0.691^{\;a}$
Smoking	12 (18.5%)	3 (25.0%)	
Lesion size (mm)	34.5 ± 19.8	21.9 ± 12.4	$0.008^{\;a}$
>20 mm	47 (72.3%)	5 (41.7%)	$0.049^{\;b}$
≤20 mm	18 (27.7%)	7 (58.3%)	
Lesion depth from pleura (mm)	12.9 ± 9.7	11.2 ± 11.2	$0.630^{\;b}$
Lesion character			
Solid lesion	61 (93.8%)	8 (66.7%)	$0.018^{\;b}$
Subsolid lesion	4 (6.2%)	4 (33.3%)	
Location			$0.344^{\;c}$
RUL	20 (30.8%)	6 (50.0%)	
RML	3 (4.6%)	2 (16.7%)	
RLL	13 (20.0%)	2 (16.7%)	
LUL	16 (24.6%)	0	
LLL	11 (16.9%)	2 (16.7%)	
Mediastinum	1 (1.5%)	0	
Chest wall	1 (1.5%)	0	
Patient position			$0.752^{\;c}$
Supine	24 (36.9%)	5 (41.7%)	
Prone	35 (53.8%)	7 (58.3%)	
Left decubitus	5 (7.7%)	0	
Right decubitus	1 (1.5%)	0	

*^a^* Fisher’s exact test, *^b^* Welch’s *t*-test, *^c^* Chi-square test of independence.

**Table 5:** Previous studies using CBCT-guided biopsy for peripheral pulmonary lesions.

Author Year	Number of Patients	Lesion Size	Operator	System	Radiation Dose	Diagnostic Accuracy	Procedure Time	Complications
Cheng et al., 2015 [27]	104 patients 35 under CBCT 69 under CCT	mean CBCT: 30 ± 14 CCT: 35 ± 19	IR	CT scanner: Brilliance 64 (Philips) CBCT: AlluraXperFD20 (Philips)	CBCT group (3.4 ± 2.1 mSv) CCT group (3.9 ± 0.79 mSv)	CBCT:Acc: 97% Sen: 97%/Spe: 100% CT:Acc: 100% Sen: 97%/Spe: 100%	CBCT group (32 ± 11 min) CCT group (38 ± 9.7 min) *p* = 0.009	CBCT: Haemoptysis: 4 Pneumothorax: 7 CCT: Haemoptysis: 5 Pneumothorax: 14
Soidan et al., 2020 [8]	CBCT 98 biopsies in 94 patients	35.6 ± 20.6 mm	IR	CBCT: Artis Zee floor (Siemens)	Total dose (milliGy) 71,567.5 ± 329.9 Area dose (microGy/m^2^) 11,722.4 ± 6681.4 (Effective dose 18.75 ± 10.6 mSv)	Sen: 91.5% Spe: 100%	Fluoroscopy time (avg each case) 4.99 min	Pneumothorax: 38 cases (38.8%) Alveolar haemorrhage: 17(17.3%) Emphysema: 4 (4.1%) Haemoptysis: 2 (2%)
Yang et al., 2022 [15]	217 patients Conventional CT: 82 patients CBCT: 135 patients		IR	CT scanner: Ingenuity Core128 (Philips) CBCT: Artis Zee (Siemens Healthcare)	Effective dose of X-ray (mSv) CCT: 13.4 (10.9–17.4) CBCT: 7.6 (6.0–9.5) *p* < 0.001	Acc: CCT: 81/82 (98.8%) CBCT: 134/135 (99.3%)	Operation time (min) CCT: 25 (21–31) CBCT: 15 (12–20) *p* < 0.001	CCT: Pneumothorax: 15 (18.3%), Pulmonary haemorrhage: 25 (30.5%) CBCT: Pneumothorax: 16 (11.9%), Pulmonary haemorrhage: 22 (16.3%)
Hwang et al., 2010 [28]	CBCT 27 patients	13 ± 4 mm	IR	CBCT: AlluraXperFD20 (Philips)	4.6 mSv (range, 2.19–9.37)	Sen: 94% (16/17) Spe: 89% (8/9) Acc: 92% (24/26)	12 ± 4 min (range, 5–22 min)	Pneumothorax: 3 Chest tube insertion: 1 Haemoptysis: 1
Ahn et al., 2019 [7]	CT 239 patients	mean: 39.5 ± 19.3 mm	IP	CT: SOMATOM (Siemens)		Sen: 96.1% Spe: 100% Acc: 96.9% (217/224)		Pneumothorax: 7.0% (67/248), Haemoptysis: 5.2% (13/248), haemothorax 0.8% (2/248)
Lee et al., 2014 [2]	1108 patients CBCT-guided PTNBs	mean size 2.7 cm ± 1.7	IR	Axiom Artis (Siemens) CBCT: AlluraXperFD20 (Philips)	7.3 mSv ± 4.1 DAP: 32,880.0 mGy⋅cm^2^ ± 28,061.6	Acc: 1148/1153 (99.6%)	Mean procedure time 14.9 min ± 6.1	Pneumothorax: 196 (17.0%), Haemoptysis: 80 (6.9%)
Fior et al., 2019 [5]	375 CBCT-CNBs biopsies	mean size 38.5 ± 25.1 mm	IR	CBCT: AlluraXperFD20 (Philips)	Mean effective radiation dose 7.12 ± 8.78 mSv	Acc: 97.2%		Pneumothorax 57 (15.2%), Perilesional haemorrhage 41 (10.9%), Haemoptysis: 9 (2.4%) procedures
Choo et al., 2012 [29]	CBCT 107 consecutive PCNBs in 105 patients	mean size, 0.85 cm ± 0.14	IR	CBCT: AlluraXperFD20 (Philips)	Estimated radiation exposure 5.72 mSv ± 4.19	Sen: 96.7% (58/60) Spe: 100% (38/38) Acc: 98.0% (96/98)	Total procedure time: 10.5 min ± 3.2	Total complications: 13 (12.1%) cases; Pneumothorax: 7 (6.5%) Haemoptysis: 6 (5.6%)
Rotolo et al., 2016 [30]	319 patients 324 TNBs CBCT vs. CCT	CBCT: 20 ± 6.5 mm CCT: 20 ± 6.8 mm	IR	CT: Aquilion 64 (Toshiba) CBCT: AlluraXperFD20 (Philips)	Mean: CBCT: 14.3 ± 10.0 CCT: 16.2 ± 10.0 mSv	CBCT: Acc: 96% Sen: 95%/Spe: 100% CT: Acc: 94% Sen: 92%/Spe: 100%		Pneumothorax—all cases, *n* (%) CBCT-guided: 36 (29.3), Fluoro CT-guided: 53 (26.7)
Jiao et al., 2015 [19]	CBCT 100 patients	mean: 1.25 cm ± 0.39 (0.50–2.00 cm)	IR	Artis zeego (Siemens)	Mean exposure dose of 7.6 mSv ± 3.1	Sen: 98.7% (77/78) Spe: 90.5% (19/21) Acc: 97.0%	The mean total procedure time was 12.84 min ± 3.74	Pneumothorax rate (%) 10 (10) Haemoptysis rate (%) 12 (12)
Jeon et al., 2018 [26]	CT-laser guidance 244 patients	mean 33.8 ± 20.1 mm	IP	CT: SOMATOM Sensation 64 (Siemens)	Mean effective radiation dose: 1.0 ± 0.4 mSv	Sen: 94.4% (152/161), Spe: 100% (77/77), Acc: 96.2% (229/238)	Total procedure time: 8.8 ± 2.9 min	Pneumothorax: 21.8% (53/243), Haemoptysis: 9.1% (22/243), Haemothorax 1.6% (4/243)
Current study	CBCT-laser guidance 77 patients	mean size 32.6 ± 19.4 mm	IP	CBCT: ARTIS pheno (Siemens)	Median total DAP 8485.4 ± 5819.9 µGym^2^ (Effective dose 13.57 ± 9.31 mSv)	Sen: 93.3% (56/60) Spe: 100% Acc: 94.8% (73/77)	Median procedure time: 21.1 min	Pneumothorax 10.4% (8/77), Haemoptysis 1.3% (1/77) and Haemothorax 1.3% (1/77)

## Data Availability

The datasets generated and analysed during this study contain potentially identifiable clinical information and cannot be made publicly available. De-identified data supporting the findings are available from the corresponding author upon reasonable request.

## Supplementary Materials

The following supporting information can be downloaded at: https://www.mdpi.com/article/10.3390/diagnostics16020226/s1. STROBE checklist.
